# Quantification of *in Vivo* Colonic Short Chain Fatty Acid Production from Inulin

**DOI:** 10.3390/nu7115440

**Published:** 2015-10-28

**Authors:** Eef Boets, Lise Deroover, Els Houben, Karen Vermeulen, Sara V. Gomand, Jan A. Delcour, Kristin Verbeke

**Affiliations:** 1 Translational Research in Gastrointestinal Disorders, KU Leuven, Leuven 3000, Belgium; eef.boets@med.kuleuven.be (E.B.); lise.deroover@med.kuleuven.be (L.D.); els.houben@uzleuven.be (E.H.); 2 Leuven Food Science and Nutrition Research Centre, KU Leuven, Leuven 3000, Belgium; sara.gomand@biw.kuleuven.be (S.V.G.); jan.delcour@biw.kuleuven.be (J.A.D.); 3 Clinical Department of Laboratory Medicine, University Hospitals Leuven, Leuven 3000, Belgium; 4 Department of Pathology, Bacteriology and Avian Diseases, Ghent University, Merelbeke 9820, Belgium; karen.vermeulen@ugent.be; 5 Centre for Food and Microbial Technology, KU Leuven, Leuven 3000, Belgium

**Keywords:** short chain fatty acids, colonic fermentation, inulin

## Abstract

Short chain fatty acids (SCFA), including acetate, propionate, and butyrate, are produced during bacterial fermentation of undigested carbohydrates in the human colon. In this study, we applied a stable-isotope dilution method to quantify the *in vivo* colonic production of SCFA in healthy humans after consumption of inulin. Twelve healthy subjects performed a test day during which a primed continuous intravenous infusion with [1-^13^C]acetate, [1-^13^C]propionate and [1-^13^C]butyrate (12, 1.2 and 0.6 μmol·kg^−1^·min^−1^, respectively) was applied. They consumed 15 g of inulin with a standard breakfast. Breath and blood samples were collected at regular times during the day over a 12 h period. The endogenous rate of appearance of acetate, propionate, and butyrate was 13.3 ± 4.8, 0.27 ± 0.09, and 0.28 ± 0.12 μmol·kg^−1^·min^−1^, respectively. Colonic inulin fermentation was estimated to be 137 ± 75 mmol acetate, 11 ± 9 mmol propionate, and 20 ± 17 mmol butyrate over 12 h, assuming that 40%, 10%, and 5% of colonic derived acetate, propionate, and butyrate enter the systemic circulation. In conclusion, inulin is mainly fermented into acetate and, to lesser extents, into butyrate and propionate. Stable isotope technology allows quantifying the production of the three main SCFA *in vivo* and proved to be a practical tool to investigate the extent and pattern of SCFA production.

## 1. Introduction

Short chain fatty acids (SCFA), such as acetate, propionate and butyrate, are produced in the colon during bacterial fermentation of undigested carbohydrates and to a lesser extent of proteins. Carbohydrate fermentation predominates in the proximal colon where substrates are abundantly available for fermentation, which explains the decline in levels of luminal SCFA when progressing towards the distal colon [1]. SCFA production from undigested carbohydrates involves different steps. First, the undigested carbohydrates are broken down into monosaccharides via microbial hydrolysis. Secondly, the monosaccharides are fermented to phosphoenolpyruvate (PEP) via the Embden-Meyerhof-Parnas pathway. Finally, acetate, propionate and butyrate are produced from PEP via different reactions. Propionate is mainly produced via the succinate pathway and the acrylate pathway. The most important pathway is the succinate pathway utilized by Bacteriodetes and Negativicutes [2]. Production of acetate and butyrate require PEP conversion into acetyl-coenzyme A (acetyl-CoA). Acetate is formed directly from acetyl-CoA by many Firmicutes. Butyrate can be produced via either butyrate kinase or butyryl-CoA:acetate CoA-transferase. The latter is considered the most important route and is only possible in the presence of acetate [3]. Well-known bacteria using this pathway are Firmicutes belonging to *Clostridium clusters* IV and XIVa [4].

After production in the colon, SCFA are rapidly and almost completely absorbed by the colonocytes (only 5%–10% is excreted in feces) where part of them, in particular butyrate, are oxidized. In this way, SCFA are important energy substrates which contribute to up to 70% of the energy requirements of the colonocytes [5]. The remaining SCFA are transported through the portal vein into the liver. Measurement of fluxes of SCFA across the gut and liver in humans undergoing abdominal surgery indicated a significant uptake of propionate and butyrate (but not of acetate) by the liver which counterbalanced the release by the gut. In particular acetate and to a minor extent propionate were released into the systemic circulation whereas no splanchnic release of butyrate was observed [6].

Several *in vitro* studies as well as experiments in different laboratory and production animals have demonstrated the impact of SCFA on mammalian physiology. In addition, it has become evident that each of the individual SCFA affects health differently. For example, whereas acetate acts as a precursor for lipogenesis and cholesterol synthesis [7,8,9], propionate has been reported to inhibit acetate incorporation into cholesterol. Indeed, acetate incorporation in cholesterol was lower in healthy humans when acetate was rectally infused in combination with propionate than when it was infused alone [10]. Similarly, anti-inflammatory effects of the SCFA depend on the type of acid. Butyrate and propionate, but not acetate, inhibit histone deacetylases (HDACs) and affect in this way the expression of various genes [11]. Inhibition of HDACs prevents activation of NF-κB, which is one of the key transcription factors that regulate the expression of genes implicated in innate immunity, cell cycle control and apoptosis [12], and in the release of inflammatory cytokines [13]. A recent cell-based screening assay based on analysis of the activity of the NF-κB pathway showed that SCFA reduce NF-κB activity in the order butyrate > propionate >> acetate [14]. More recently, it was shown that inhibition of HDACs by butyrate and propionate induces the immunosuppressive enzymes indoleamine-2,3-dioxygenase (IDO1) and aldehyde dehydrogenase 2 (Ald1A2) in dendritic cells. This potentiates their ability to convert naïve T cells into FoxP3+ regulatory T cells and to suppress the conversion of naïve T cells into INF-γ + T cells [15]. In addition, the interaction of SCFA with G-protein coupled receptor (GPR) 43, also known as free fatty acid receptor (FFAR) 2, profoundly affects inflammatory processes which might explain the anti-inflammatory effect of acetate. In mice, stimulation of GPR43 by SCFAs was necessary for the normal resolution of inflammatory responses, as GPR43-deficient (Gpr43^−/−^) mice showed exacerbated or unresolving inflammation in models of colitis, arthritis, and asthma [16].

Activation of GPR43 (FFAR2) as well as of GPR41 (FFAR3) by SCFA has also been postulated as a mechanism by which SCFA regulate energy homeostasis. The selectivity of the SCFA for both receptors depends on their chain length. This explains the differential effects of each SCFA, with butyrate being more selective for GPR41, acetate more selective for GPR43, and propionate binding to both receptors [17]. In addition, propionate and butyrate, but not acetate, may activate intestinal gluconeogenesis (IGN), albeit by a different mechanism, leading to increased glucose levels in the portal vein. Butyrate acts through a cAMP-dependent mechanism, whereas propionate, itself a substrate of IGN, activates IGN gene expression via a gut-brain neural circuit involving GPR41. The increased glucose levels are sensed by a glucose sensor that transmits the signal to the brain by the peripheral nervous system to promote beneficial effects on food intake and glucose metabolism [18]. In contrast, propionate is the only SCFA that can be used as a precursor for gluconeogenesis in the liver [19,20]. Recently, a GPR-independent mechanism, common to each of the SCFA has been revealed. SCFA supplementation in mice down regulated peroxisome proliferator-activated receptor-γ (PPAR-γ) activity, resulting ultimately in a shift of the metabolism in adipose and liver tissue from lipogenesis to fatty acid oxidation [21].

In humans, evidence for the beneficial physiological effects of SCFA is more scarce. A major reason is the lack of reliable information on *in vivo* production and absorption kinetics of the individual SCFA. In view of the different mechanisms by which individual SCFA exert their physiological effects, it might well be important to not only know the total amount of SCFA formed, but also their relative proportions. Quantification of the *in vivo* colonic SCFA production is rather difficult due to the inaccessibility of the production site and the rapid absorption by the colonocytes. An elegant strategy to circumvent these difficulties is the use of stable isotope dilution. In this technique, a constant intravenous infusion of ^13^C-labeled SCFA is applied which results in a constant ^13^C-SCFA enrichment in blood. During fermentation of an undigestible carbohydrate in the colon unlabeled SCFA are produced that enter the circulation and dilute the ^13^C-SCFA, resulting in a decreased ^13^C-SCFA enrichment. In the present study we used stable isotope dilution to quantify acetate, propionate, and butyrate production *in vivo* in the human colon during fermentation of inulin as a model substrate.

## 2. Materials and Methods

### 2.1. Study Population

Twelve healthy men and women aged 18–65 year were included in the study. Inclusion criteria included a body mass index between 18.5 and 28.5 kg/m^2^ and a regular diet with three meals a day, at least five times a week. Exclusion criteria were a history of metabolic or gastrointestinal disease or former abdominal surgery (except for appendectomy), the use of antibiotics or any other medical treatment influencing gut transit or intestinal microbiota for at least three months, consumption of a low calorie or other special diet during the last month prior to the study, pregnancy or breastfeeding, diabetes (type 1 and 2), and hemoglobin (Hb) levels below reference values. The study protocol complied with the Helsinki Declaration and was approved by the Ethics Committee of the University of Leuven. All participants gave written informed consent. The study was registered at ClinicalTrial.gov (clinical trial number: NCT01757379).

### 2.2. Study Design

During the three days prior to the test day the subjects consumed a low fiber diet (consisting of maximum one piece of fruit and 100 g vegetables a day, and white flour instead of wholemeal products) and avoided alcohol consumption. On the evening before the test day a completely digestible and non-fermentable meal (lasagna) was consumed. After an overnight fast, the subjects presented at the laboratory and provided two basal breath samples for measurement of H_2_ and ^14^CO_2_. In both arms a catheter was introduced into an antecubital vein. One catheter was used to collect blood samples and via the second catheter a primed continuous infusion of ^13^C-labeled SCFA (sodium [1-^13^C]acetate: 6 μmol·kg^−1^ + 12 μmol·kg^−1^·h^−1^; sodium [1-^13^C]propionate: 0.6 μmol·kg^−1^ + 1.2 μmol·kg^−1^·h^−1^; sodium [1-^13^C]butyrate: 0.3 μmol·kg^−1^ + 0.6 μmol·kg^−1^·h^−1^) (99% ^13^C enrichment, Euriso-top, St. Aubin, Cédex, France) was administered. After collection of a basal blood sample, the infusion was started and the subjects received a standard breakfast (pancake; 8.4 g protein, 26.7 g carbohydrate, 11.2 g fat, 244 kcal) together with 15 g inulin (Raftilin HP (degree of polymerisation ranging from 2 to 60 with an average of 23) Beneo-Orafti, Mannheim, Germany) dissolved in 200 mL of water. To determine the time of arrival of the meal in the colon, inulin-^14^C-carboxylic acid (74 kBq, ARC, St. Louis, MO, USA) was added to the breakfast. After breakfast, breath samples were collected every 20 min for up to 10 h. Blood samples were collected every hour during the first 4 h, every 20 min from 4 to 9 h and every 40 min from 9 to 12 h. After 4 h and 8 h, the subjects received a standard, completely digestible meal (white bread with ham or cheese). Finally, all subjects delivered a fecal sample that was stored at 4 °C at home until it could be delivered in the laboratory where it was frozen at −80 °C within 10 h after collection until analysis.

### 2.3. Analytical Procedures

#### 2.3.1. Analysis of Breath Samples

Breath samples for hydrogen analysis were collected in Exetainers^©^ (Labco Ltd., Ceredigion, UK) and analyzed using a hydrogen monitor (M.E.C., Brussels, Belgium). Hydrogen excretion was expressed in parts per million (ppm). A significant increase in H_2_ in breath was defined as an increase of 2.5 times the standard deviation of all previous points above the running average of all previous points [22]. Breath samples for analysis of ^14^CO_2_ were collected by blowing through a pipette into a vial containing 2 mmol hyamine hydroxide until discoloration of the thymolphtaleine indicator, corresponding to the capture of 2 mmol CO_2_. The amount of ^14^CO_2_ was measured using β-scintillation counting (Packard Tricarb Liquid Scintillation Spectrometer, model 3375; Packard Instruments, Downers Grove, IL, USA) after addition of 10 mL of Hionic fluor (Perkin Elmer, Boston, MA, USA) and expressed as disintegrations per minute (dpm). The time of arrival of the meal in the colon was defined as the time at which a significant increase in ^14^CO_2_ was observed in the breath and was determined in a similar way as for H_2_.

#### 2.3.2. Analysis of ^13^C Enrichment in Plasma Samples

Venous blood was sampled into EDTA tubes (BD Vacutainer^®^, Erembodegem, Belgium) and centrifuged to obtain plasma which was aliquoted and frozen at −80 °C. Plasma samples were thawed prior to analysis and prepared as described by Morrison *et al.* [23]. The ^13^C/^12^C ratio of SCFA was measured on a Delta plus-XP isotope ratio mass spectrometer (GC-C-IRMS, Thermo Fisher, Bremen, Germany) equipped with a trace gas chromatograph (Interscience, Breda, The Netherlands) and a combustion interface type 3 (Thermo Fisher). Separation of the SCFA was achieved with an AT-Aquawax-DA column (30 m × 0.53 mm, i.d., 1.00 μm, Grace, Lokeren, Belgium) on which 4 μL was injected splitless with the injector temperature at 240 °C. Helium 5.0 was used as carrier gas, at a constant flow of 2.5 mL/min. The oven temperature was first kept at 80 °C for 3 min, then ramped to 140 °C at 4 °C/min, further increased to 240 °C at 16 °C/min and then held for 1 min. The separated GC-effluents were online combusted to NO*_x_*, CO_2_, SO_2_, (SiO_2_)*_x_*, and H_2_O in an oxidation furnace (CuO/NiO/Pt) at 940 °C. NO*_x_* was reduced to N_2_ and, in addition, O_2_ bleed from the oxidation oven was removed by the reduction reactor operating at 640 °C. The produced water was removed by an online Nafion capillary (Thermo Fisher). The delta (δ^13^PDB) values were calculated using Isodat 2.0 software (Thermo Fisher) and expressed as atom percentage (AP). At any time point t, the measured AP was corrected for the baseline AP by subtracting it with the enrichment measured in the baseline sample which results in the atom per cent excess (APE) [24].

#### 2.3.3. Analysis of Butyrate Producing Capacity in Fecal Samples

Quantitative PCR (qPCR) was used to quantify the abundance of *Clostridium* cluster IV, *Clostridium* cluster XIV, butyryl-CoA:acetate-CoA transferase and butyrate kinase genes in fecal samples using primers described elsewhere [25,26,27,28]. DNA was extracted from fecal samples using the CTAB (cetyltrimethylammonium bromide) method, as previously described by Griffiths *et al.* [29]. To 200 mg of fecal sample, 0.5 g of unwashed glass beads (Sigma-Aldrich, St. Louis, MO, USA), 0.5 mL of CTAB buffer (5.0% (w/v), 0.35 M NaCl, 120 mM K_2_HPO_4_) and 0.5 mL of phenol-chloroform-isoamyl alcohol (25:24:1) (Sigma-Aldrich, St. Louis, MO, USA) were added, followed by homogenization in a 2 mL destruction tube using a beadbeater (MagnaLyser, Roche, Basel, Switzerland). After centrifugation (10 min, 5900 *g*), 300 μL of the supernatant was transferred to a new Eppendorf tube. For a second time, 0.25 mL of CTAB buffer was added to the original DNA sample and 300 μL of the supernatant was added to the first 300 μL. The phenol was removed by mixing with an equal volume of chloroform-isoamyl alcohol (24:1) (Sigma-Aldrich, St. Louis, MO, USA) followed by centrifugation (10 s, 11,700 *g*) at room temperature. Total nucleic acids were subsequently precipitated from the extracted aqueous layer with two volumes of PEG-6000 solution (polyethyleenglycol 30% (w/v), 1.6 M NaCl) (Fluka BioChemika, Sigma-Aldrich, Bornem, Belgium) for 2 h at room temperature. After centrifugation (20 min, 9500 *g*), the pellet was rinsed with 1 mL of ice-cold 70% (v/v) ethanol and air dried prior to suspension in 100 μL RNase free water (VWR, Leuven, Belgium).

Amplification and detection (CFX96 Biorad Detection System, Biorad, Nazareth-Eke, Belgium) were carried out using 2× Sensimix^TM^ SYBR No-ROX mix (Bioline, Kampenhout, Belgium). Each reaction was done in triplicate in a 12.0 μL total reaction mixture using 2.0 μL of appropriate dilutions of the DNA sample and 0.5 μM (*Clostridium* cluster IV), 0.2 μM (*Clostridium* cluster XIV), 2.5 μM (butyryl-CoA:acetate-CoA transferase), 0.4 μM (butyrate kinase) final quantitative PCR primer concentration. The amplification program consisted of 1 cycle of 95 °C for 10 min, followed by 40 cycles of 95 °C for 30 s, 60 °C for 1 min (*Clostridium* cluster IV), 40 cycles of 95 °C for 30 s, 52 °C for 1 min (*Clostridium* cluster XIV), 40 cycles of 95 °C for 30 s, 53 °C for 30 s, 72 °C for 30 s (butyryl-CoA:acetate-CoA transferase), and 40 cycles of 95 °C for 30 s, 60 °C for 1 min, 72 °C for 30 s (butyrate kinase). A stepwise increase of the temperature from 55 to 95 °C (at 5 s/0.5 °C) was added to analyze melting curve data to confirm the specificity of the reactions.

PCR primers and DNA used for construction of the standard curves are listed in Table 1. After purification and determination of the DNA concentration, the volume of the linear double-stranded DNA standard was adjusted to 6.04 × 10^8^ copies·mL^−1^ assuming an average molecular weight of 660 per nucleotide pair. This stock solution was 10-fold serially-diluted to obtain a standard series from 6.04 × 10^7^ to 6.04 × 10^1^ copies·mL^−1^. The copy numbers of samples were determined by reading off the standard series with the Ct values of the samples. Gene copy numbers were expressed as log_10_ values per gram wet weight of feces.

**Table 1:** Primers used for construction of standard curves for quantification of the butyrate producing capacity present in fecal samples.

Standard Curve	DNA Source	Oligonucleotide	Sequence (5′–3′)
*Clostridium* Cluster IV	*Butyricicoccus pullicaecorum*	Forward primer	AGTACGGCCGCAAGGTTGAAA
		Reverse primer	CTGCCATTGTAGTACGTGTG
*Clostridium* Cluster XIV	*Butyricicoccus pullicaecorum*	Forward primer	TGACCGGCCACATTGGGACTG
		Reverse primer	TCATCCCCACCTTCCTCCAG
Butyryl-CoA:acetate-CoA transferase	*Butyricicoccus pullicaecorum*	Forward primer	AATCCGGAGACTGGGTAGAT
		Reverse primer	GGACAGATAAGCTCCGAGC
Butyrate kinase	*Clostridium perfringens*	Forward primer	TGGGGGAGGAAAGTTATATGGC
		Reverse primer	CTCCTACTGAAACTCCGCCC

### 2.4. Calculations

In this study, it was assumed that the rate of SCFA appearance and that of the infusion of ^13^C-labeled SCFA enter into a single, homogenous, instantly-mixing pool from which sampling occurs. The rate of appearance (R_a_; μmol·kg body weight^−1^·min^−1^) of acetate, propionate, and butyrate at each time point was calculated using the rate of infusion of ^13^C-SCFA (i), the isotopic enrichment of the infused SCFA (tracer enrichment), and the isotopic enrichment of the SCFA measured in plasma (plasma enrichment) according to Equation (1) [30]:

(1) $${\text{R}}_{a}=i\;x\;\left[\left(\frac{Tracer\;enrichment}{Plasma\;enrichment}\right)-1\right]$$

with tracer and plasma enrichment expressed in atom percent excess (APE).

The endogenous R_a_ reflects the rate of SCFA appearance in the absence of colonic fermentation from the administered substrate. It was calculated from the mean plateau enrichment obtained during the first 3 h of the infusion. Endogenous R_a_ was subtracted from the whole body R_a_ to obtain the increase in SCFA originating from colonic fermentation of inulin (exogenous SCFA production). The cumulative recovery during the 12 h observation period was determined by calculating the area under the curve (AUC) of the exogenous R_a_ of the SCFA-time curve using the trapezoidal method and was multiplied by the body weight to yield total amount of SCFA recovered (in μmol) in plasma.

To estimate colonic production, total amounts of SCFA recovered in plasma during the 12 h observation period were multiplied by a bioavailability index that accounts for the extraction of the SCFA in the splanchnic bed [31]. Based on literature data, we assumed mean bioavailability indices of 40% [30], 10% [32] and 5% [6,33] for acetate, propionate, and butyrate, respectively. The bioavailability index of butyrate was based on the observation that the colonocytes remove approximately 90% [33,34] of butyrate and that subsequently more than 50% of the remaining butyrate is extracted by the liver [6].

### 2.5. Statistics

Statistical analyses were performed by using SPSS, version 22.0 (IBM, Brussels, Belgium). All results are presented as mean ± standard deviation. Normality was checked with the Shapiro-Wilk protocol. All analyses were performed using linear mixed models, paired *t*-tests or Pearson’s product-moment correlation coefficients, except for correlations including colonic butyrate production, whole-body R_a_ of propionate, and butyrate, which were analyzed using Spearman’s rho correlation coefficients. Pairwise comparisons using paired samples *t*-tests were corrected with false discovery rate (FDR) for multiple testing.

## 3. Results

### 3.1. Study Population

In total, 17 interested subjects attended a screening examination that included assessment of height and body weight, plasma hemoglobin levels, medical history and filling out a dietary questionnaire. Three subjects dropped out due to lack of time and two candidates were excluded because of hemoglobin levels below the reference values. Thus, 12 subjects performed the test day according to the protocol. Baseline characteristics are presented in Table 2.

**Table 2:** Baseline characteristics of the 12 healthy subjects who completed the study.

	Men	Women	*p* Value
*N*	5	7	
Age (year)	27 ± 8	24 ± 4	0.530
Length (m)	1.82 ± 0.09	1.65 ± 0.06	0.003
Weight (kg)	78 ± 8	57 ± 4	0.005
Body mass index (kg/m^2^)	24 ± 4	21 ± 2	0.149
log butyrate kinase (copies/g feces)	3.83 ± 0.79	3.97 ± 0.80 *	0.792
log butyryl-CoA:acetate-CoA transferase (copies/g feces)	8.14 ± 0.49	7.85 ± 0.66 *	0.429
log *Clostridium* cluster IV (copies/g feces)	8.80 ± 0.58	8.67 ± 0.60 *	0.662
log *Clostridium* cluster XIV (copies/g feces)	9.72 ± 0.31	9.52 ± 0.47 *	0.537

* *n* = 6, one female subject was not able to deliver a sample.

### 3.2. Steady-State Characteristics

At baseline, hydrogen excretion in breath was lower than 15 ppm in all subjects and remained unchanged up to about 240 min after breakfast. Endogenous R_a_ of SCFA was determined based on the plasma enrichments measured during the first three hours after inulin ingestion. No fermentation of inulin was observed during this period based on the breath hydrogen results implicating that no SCFA were produced in the colon and no SCFA were entering the plasma. Endogenous R_a_ was significantly higher for acetate compared to propionate and butyrate (*p* < 0.001, Table 3).

**Table 3:** The enrichments and rate of appearances (R_a_) of acetate, propionate, and butyrate in plasma in healthy subjects before and during inulin fermentation. (APE: atom percent excess).

		Acetate	Propionate	Butyrate	*p*-Value
Isotopic enrichment (APE)	*Before inulin fermentation*	0.86 ± 0.36 ^a^	2.53 ± 0.74 ^b^	1.03 ± 0.47 ^a^	< 0.001
	*Minimum during fermentation*	0.48 ± 0.20 ^c^	1.60 ± 0.59 ^d^	0.61 ± 0.27 ^c^	< 0.001
R_a_ (μmol·kg^−1^·min^−1^)	*Before inulin fermentation*	13.26 ± 4.82 ^e^	0.27 ± 0.09 ^f^	0.28 ± 0.12 ^f^	< 0.001
	*Maximum during fermentation*	24.98 ± 10.70 ^g^	0.47 ± 0.23 ^h^	0.50 ± 0.29 ^h^	< 0.001

All values expressed as mean (±SD), *n* = 12. Measurements were statistically evaluated using linear mixed models and *p*-values refer to overall significance of the linear mixed model. Different letters in superscript indicate significant differences after pairwise comparisons using paired samples *t*-tests with false discovery rate (FDR) correction for multiple testing, *p* < 0.01.

**Figure 1:**
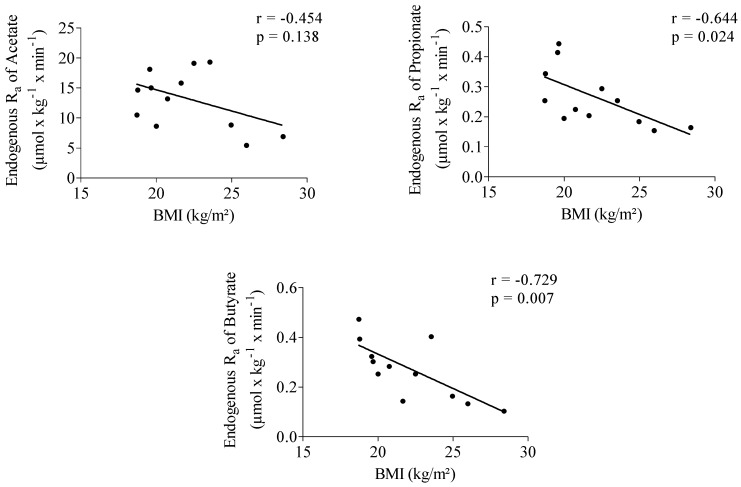
Correlation between acetate, propionate and butyrate endogenous rate of appearance (R_a_) and body mass index (BMI). *n* = 12.

Endogenous R_a_ of propionate showed a significantly positive correlation with acetate (*r* = 0.652, *p* = 0.022). No correlation was observed between the endogenous R_a_ of butyrate and acetate (*r* = 0.478, *p* = 0.116) and between R_a_ of butyrate and propionate (*r* = 0.551, *p* = 0.063). Propionate (*r* = −0.644, *p* = 0.024) and butyrate (*r* = −0.729, *p* = 0.007) endogenous R_a_ were significantly negatively correlated with body mass index (BMI) (Figure 1).

### 3.3. Impact of Inulin on Rate of Appearance of SCFA

#### 3.3.1. Start of Fermentation

After arrival into the colon of the undigested part of the breakfast labeled with inulin-^14^C-carboxylic acid, the latter is fermented resulting in the production of ^14^CO_2_ which is excreted in breath. Arrival in the colon, thus indicated by the increased excretion of ^14^CO_2_ in breath, occurred 364 ± 87 min after consumption of the breakfast and was not significantly different (*p* = 0.094) from the time point of increased hydrogen excretion in breath (338 ± 94 min) indicating the start of fermentation of the unlabeled inulin.

#### 3.3.2. SCFA Enrichment in Plasma and Rate of SCFA Appearance

**Figure 2:**
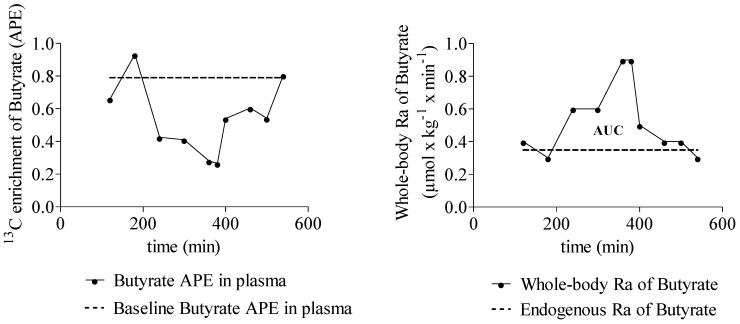
Typical example that shows the ^13^C enrichment (APE) of butyrate in plasma over time and the whole-body rate of appearance (Ra) of butyrate over time. *n* = 1.

The R_a_ of SCFA increased significantly during fermentation of inulin (Table 3). A representative example of the ^13^C enrichment of butyrate and whole body R_a_ of butyrate in one subject is shown in Figure 2. A positive correlation was observed between whole-body R_a_ of propionate and butyrate (*r* = 0.657, *p* = 0.020). No correlations were observed between butyrate and acetate whole-body R_a_ and between propionate and acetate whole-body R_a_.

#### 3.3.3. Quantification of SCFA Production from Inulin

Twelve hours after consumption of the inulin, the SCFA R_a_ of all subjects had returned to the endogenous R_a_ level. The cumulative amounts of exogenous SCFA production appearing in plasma amounted to 55 ± 30, 1.1 ± 0.9, and 1.0 ± 0.9 mmol for acetate, propionate, and butyrate, respectively (Table 4). The estimated colonic production was 137 ± 75 mmol for acetate, 11 ± 9 mmol for propionate, and 20 ± 17 mmol for butyrate over the 12 h period after inulin ingestion (Table 4). Production of SCFA was not related to the BMI of the subjects (*p* = 0.778, 0.749 and 0.633 for acetate, propionate, and butyrate, respectively).

**Table 4:** Cumulative amount (AUC) of exogenous short chain fatty acid (SCFA) production, SCFA in the peripheral circulation after consumption of 15 g of inulin in healthy subjects, and calculation of the amounts produced in the colon.

	Acetate	Propionate	Butyrate
Cumulative amount SCFA in plasma (μmol/kg)	860 ± 497	17 ± 13	16 ± 15
Peripheral SCFA (mmol)	55 ± 30	1.1 ± 0.9	1.0 ± 0.9
Colonic SCFA (mmol) *	137 ± 75	11 ± 9	20 ± 17
Ratio (%) **	82	6	12

* Calculated based on literature data assuming that a constant percentage of colonic derived acetate (40% [27]), propionate (10% [29]), and butyrate (5% [3,30]) appear in plasma; ** The ratio indicates the contribution of acetate, propionate and butyrate to the total amount of colonic produced SCFA expressed as molar ratio.

#### 3.3.4. Butyrate-Producing Capacity

**Figure 3:**
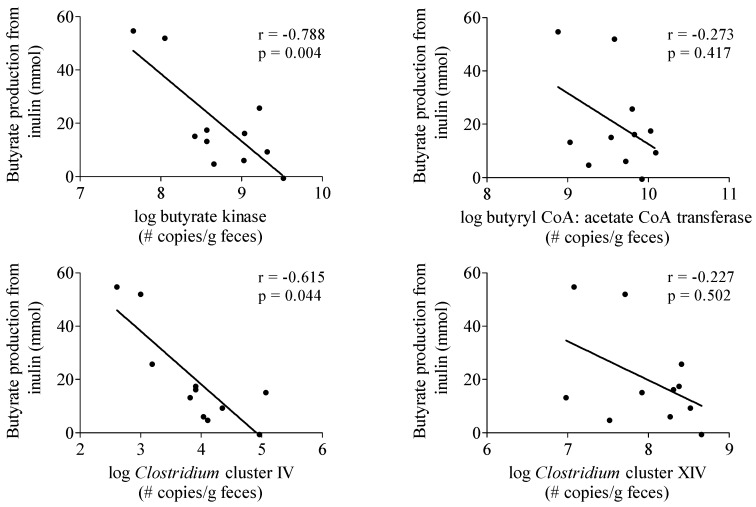
Correlation between butyrate production from inulin and parameters of butyrate producing capacity. *n* = 11, one subject was not able to deliver a sample.

To investigate whether the production of butyrate from inulin depended on the intestinal microbiota composition of the subjects, the butyrate-producing capacity in a fecal sample obtained from each subject was determined (Table 2). No correlation was observed between butyrate production from inulin and butyrate butyryl-CoA:acetate CoA-transferase and *Clostridium* cluster XIVa genes (Figure 3). In contrast, butyrate production was negatively correlated with butyrate kinase (*r* = −0.788, *p* = 0.004) and *Clostridium* cluster IV genes (*r* = −0.615, *p* = 0.044) (Figure 3).

## 4. Discussion

Much of the currently available information on SCFA production has been obtained from *in vitro* experiments. The simplest experimental setups used fecal microbiota as inoculum in anaerobic batch fermentations, whether or not pH controlled [35,36]. However, these conditions do not adequately mimic the *in vivo* situation as fecal microbiota do not represent the microbiota in the proximal colon [37]. In addition, fermentation products accumulate in the medium and may affect ongoing reactions. More sophisticated models include the Simulator of the Human Intestinal Microbial Ecosystem (SHIME^®^, University of Ghent, Gent, Belgium) [38,39] that harbors a microbial community resembling that from the human colon both in fermentation activity and composition. The TNO intestinal models TIM-1 and TIM-2 (TNO, Zeist, The Netherlands) consist of vessels connected with flexible walls to allow simulation of peristalsis. In addition, the vessels are equipped with a hollow fiber membrane to absorb water and SCFA [40]. In human intervention studies, fecal SCFA concentrations have often been measured as an approximation of the *in vivo* SCFA production [41]. However, this does not provide a true reflection of SCFA production since SCFA are absorbed and utilized by the colon epithelial cells. Similarly, plasma SCFA concentrations are the net result of production, absorption, and splanchnic extraction of the SCFA and do not adequately reflect colonic generation either. The use of stable isotopes provides an attractive alternative to quantify *in vivo* SCFA production. Stable isotope dilution techniques have been used in the past to study acetate kinetics in animals and humans [30,42,43].

In this study, we have shown that such approaches can also be applied for quantifying propionate and butyrate production. Indeed, they allow calculating total R_a_ of SCFA which, in steady state conditions, equals the rate of SCFA disappearance from the pool by either uptake in the tissues or by excretion in urine or other routes [44]. The total R_a_ of SCFA is composed of the amount of SCFA that is produced in the human body (exogenous and endogenous) and the amount of SCFA infused in a single pool. To determine the whole-body SCFA R_a_, as used in the present study, the tracer infusion rate was subtracted from the total R_a_ of SCFA. We assumed no contribution from colonic fermentation to the whole-body SCFA R_a_ to happen during the first 3–4 h of the test day, as the subjects had consumed a low fiber diet for the three previous days and a fiber free dinner on the evening prior to the test. Indeed, breath hydrogen levels were below 15 ppm in all subjects for up to 4 h.

Endogenous R_a_ of acetate obtained in this study are slightly higher than in previously published studies in humans (6.0–11.4 μmol·kg^−1^·min^−1^) [23,30,31,45]. Morrison *et al.* found higher endogenous acetate R_a_ (22.5 μmol·kg^−1^·min^−1^) in two subjects after repeated oral doses of a tracer solution containing both [1-^13^C]acetate and [^2^H_3_]acetate to mimic an intravenous infusion. Only one study has previously reported endogenous R_a_ of propionate. In four healthy subjects after intravenous infusion of both 1-[^13^C]-propionate and [^2^H_5_]-propionate, they found values of 17.6 μmol·kg^−1^·h^−1^ and 17.5 μmol·kg^−1^·h^−1^, respectively [46]. Our results of 0.27 μmol·kg^−1^·min^−1^ or (expressed differently as) 16.2 μmol·kg^−1^·h^−1^ are in good agreement with those results. To our knowledge, no information is available on the kinetics of butyrate in humans. Pouteau *et al.* measured endogenous R_a_ of acetate, propionate, and butyrate in rats and found a butyrate R_a_ that was 96 times lower than acetate and 13 times lower than propionate R_a_ [47]. In our study, the R_a_ of butyrate was very similar to that of propionate and only 47 times lower than that of acetate.

To convert plasma R_a_ of SCFA into the amount of SCFA produced in the colon, the exogenous R_a_ of SCFA *versus* time curves were integrated to yield total amounts of SCFA in plasma and multiplied by a bioavailability index that accounts for the extraction of the SCFA in the splanchnic bed [31]. Based on literature data, we used mean bioavailability indices of 40%, 10%, and 5% for acetate, propionate and butyrate, respectively. In this way, we calculated that colonic fermentation of inulin per gram of substrate yields 9.13 mmol of acetate, 0.72 mmol of propionate, and 1.31 mmol of butyrate which is in the same range as results from previous *in vitro* studies. Yet, these *in vitro* studies showed a broad range in amounts of SCFA produced from inulin ranging from 1.7 to 26.2 mmol/g carbohydrate for acetate, 0.1–14.7 mmol/g carbohydrate for propionate, and 0.18–11.48 mmol/g carbohydrate for butyrate [35,36,48,49,50,51,52,53]. Using the same bioavailability index for acetate, Pouteau *et al.* calculated a colonic production of 7 mmol of acetate per gram of administered lactulose [31].

Proportions of acetate:propionate:butyrate (82:6:12) confirm previous results indicating that fermentation of inulin-type fructans results in a relatively higher production of acetate compared to other indigestible carbohydrates, such as resistant starch, polydextrose, arabinogalactan, and arabinoxylan [54,55]. Remarkably, most subjects (8 out of 12) favored butyrate production over propionate production, whereas most *in vitro* studies report higher propionate than butyrate levels [35,49,50,53]. Nevertheless, some *in vitro* batch fermentation studies with inulin showed higher butyrate than propionate production [36,48]. In addition, fermentation of inulin (degree of polymerization ranging from 2 to 60 with an average of 10) in the TIM-2 model also yielded a higher proportion of butyrate compared to propionate [55].

We observed considerable inter-individual variation in the R_a_ of SCFA which is in agreement with other studies reporting large variability in plasma SCFA concentrations in humans [6,56]. A factor that might explain this variability is the composition of the intestinal microbiota. In particular butyrate production depends on cross-feeding, *i.e.* acetate conversion into butyrate, and the presence of specific butyrate producing bacteria, mainly belonging to the *Clostridium* clusters IV and XIVa [57]. In view of the high phylogenetic diversity in human butyrate producers, functionall-based approaches have been developed. Genes involved in the pathways of butyrate production such as the butyryl-CoA:acetate CoA transferase and butyrate kinase gene can be amplified using degenerate primers that recognize multiple phylogenetic groups [26]. Surprisingly, the amount of butyrate produced from inulin, as estimated from the R_a_ of butyrate in plasma, was not positively correlated to any of the parameters of butyrate producing capacity. The relatively low number of subjects might, at least partially, explain the lack of positive correlation. However, these results may also indicate that parameters other than microbiota composition, such as absorption and splanchnic extraction of the SCFA, have a more profound impact on the R_a_ of butyrate in plasma.

Some, but not all, studies report higher fecal SCFA levels in obese subjects compared to normal weight persons [58,59,60]. Higher fecal SCFA levels have been related to higher colonic production and an increased energy harvest from undigested carbohydrates in the obese [61]. In contrast, several animal studies indicate that treatment with SCFA can reduce or reverse gains in body weight and adiposity [18,21,62]. In the present study, no increase in SCFA production in subjects with higher BMI could be confirmed. However, we found a significant negative correlation between endogenous R_a_ of propionate and butyrate with BMI, indicating that the rate of appearance of those acids in plasma is higher in subjects with lower BMI. Although plasma propionate and butyrate mainly originate from colonic fermentation, this does not necessarily point at a higher colonic production of those SCFA in low BMI subjects as also the absorption by the colonocytes and the splanchnic extraction may depend on BMI. R_a_ of acetate, which is also produced endogenously during fatty acid oxidation and glucose or amino acid metabolism, was not related to BMI. It needs to be mentioned that the BMI of the participants varied between 18.5 and 28.5 with no obese subjects participating in the study.

The major limitation of this study is the uncertainty about the SCFA bioavailability, *i.e.*, the fraction of SCFA produced in the colon that reaches the systemic circulation. Due to a lack of individual data, we relied on estimates for bioavailability reported in literature and used the same value for each individual. Stable isotope studies might also be useful to quantify SCFA bioavailabilities; for example, by introducing known amounts of labeled SCFA in the colon and quantifying the resulting labeled SCFA in the plasma, and to evaluate the impact of possible confounding factors as BMI and microbiota composition.

## 5. Conclusions

In the present study, we used a stable-isotope dilution technique to quantify the production of the three major SCFA in the colon of healthy subjects after consumption of inulin as a model substrate. However, the method is easily applicable to any other dietary fiber that is fermented into SCFA. Quantification of the amounts of total SCFA and of the proportion of the individual SCFA produced *in vivo* from different dietary fibers will facilitate the further evaluation of health benefits attributed to SCFA.
